# Mechanisms of resistance to chemotherapeutic and anti-angiogenic drugs as novel targets for pancreatic cancer therapy

**DOI:** 10.3389/fphar.2013.00056

**Published:** 2013-04-30

**Authors:** Anna Tamburrino, Geny Piro, Carmine Carbone, Giampaolo Tortora, Davide Melisi

**Affiliations:** ^1^Digestive Molecular Clinical Oncology Research Unit, Section of Medical Oncology, Department of Medicine, Università degli studi di VeronaVerona, Italy; ^2^Laboratory of Oncology and Molecular Therapy, Department of Medicine, Università degli studi di VeronaVerona, Italy

**Keywords:** pancreatic cancer, drug resistance, NF-κB, TAK-1, anti-angiogenic therapy

## Abstract

Pancreatic cancer remains one of the most lethal and poorly understood human malignancies and will continue to be a major unsolved health problem in the 21^st^ century. Despite efforts over the past three decades to improve diagnosis and treatment, the prognosis for patients with pancreatic cancer is extremely poor with or without treatment, and incidence rates are virtually identical to mortality rates. Although advances have been made through the identification of relevant molecular pathways in pancreatic cancer, there is still a critical, unmet need for the translation of these findings into effective therapeutic strategies that could reduce the intrinsic drug resistance of this disease and for the integration of these molecularly targeted agents into established combination chemotherapy and radiotherapy regimens in order to improve patients’ survival. Tumors are heterogeneous cellular entities whose growth and progression depend on reciprocal interactions between genetically altered neoplastic cells and a non-neoplastic microenvironment. To date, most of the mechanisms of resistance studied have been related to tumor cell-autonomous signaling pathways. However, recent data suggest a putative important role of tumor microenvironment in the development and maintenance of resistance to classic chemotherapeutic and targeted therapies. This present review is meant to describe and discuss some of the most important advances in the comprehension of the tumor cell-autonomous and tumor microenvironment-related molecular mechanisms responsible for the resistance of pancreatic cancer to the proapoptotic activity of the classic chemotherapeutic agents and to the most novel anti-angiogenic drugs. We present some of the emerging therapeutic targets for the modulation of this resistant phenotype.

## INTRODUCTION

Despite efforts over the past three decades to improve diagnosis and treatment, pancreatic cancer remains one of the most lethal and poorly understood human malignancies. It ranks fourth in the leading causes of cancer-related mortality among adults in Western countries (Siegel et al., 2012). The poor prognosis for patients with pancreatic cancer could be mainly attributed to the early metastatic behavior demonstrated along the progression of the disease, its aggressive course, and the limited efficacy of available systemic treatments (Vaccaro et al., 2011).

The vast majority of patients are diagnosed with locally advanced unresectable or metastatic disease, and only 15–20% of patients are eligible for initial resection (Gillen et al., 2010). In patients who undergo surgery and post-operative therapy relapse remains common and no more than 20% of patients achieve long term survival (Neoptolemos et al., 2010). For patients with advanced disease, the overall 5-years survival rate is less than 1%.

At any of these stages, pancreatic cancer has a very poor response to systemic treatments [reviewed in single topic issue (Melisi and Budillon, 2012)]. The main obstacle to the clinical efficacy of cancer treatments is the pre-existence of or the development of cellular drug resistance (DeVita and Chu, 2008), and pancreatic cancer obeys this rules.

The proposed molecular mechanisms responsible for resistance of pancreatic cancer to current treatments range from tumor cell-intrinsic mechanisms, such as activation of anti-apoptotic signaling pathways, to extrinsic mechanisms, such as the role of stromal cell compartment in drug uptake and activation of alternative escape pathways (El Maalouf et al., 2009).

In this review we discuss the rationale behind some of the tumor cell-autonomous and tumor microenvironment-related molecular mechanisms responsible for the resistance of pancreatic cancer to the pro-apoptotic activity of classic chemotherapeutic agents and to the most novel anti-angiogenic drugs.

## PANCREATIC CANCER RESISTANCE TO CLASSIC CHEMOTHERAPEUTIC AGENTS

Although recent meta-analyses suggest an advantage for chemotherapy over best supportive care for patients affected by advanced pancreatic cancer (Sultana et al., 2007), current systemic treatments offer only a modest benefit in tumor-related symptoms and survival. Over the past decade, gemcitabine has been considered to be the reference treatment in advanced pancreatic cancer. After its approval, a number of randomized controlled trials have been conducted pitting several cytotoxic and targeted agents against, or in combination with gemcitabine and none of these studies showed a clinically significant survival benefit compared with gemcitabine as single agent treatment (Giuliani et al., 2012).

Only very recently, the combination of gemcitabine with the new taxane nab-paclitaxel (Von Hoff et al., 2012), or FOLFIRINOX (Conroy et al., 2011) – a three-drug combination regimen not including gemcitabine – were able to provide a survival improvement over gemcitabine in monotherapy. Although these results represent the first steps forward in many decades of clinical research for this disease, during the treatment pancreatic cancer become invariably resistant to these polychemotherapeutic regimens so that median overall survival for metastatic patients remains less than 1 year.

Recent data suggest a critical role of tumor microenvironment in the development and maintenance of resistance to therapies. Cancer-related inflammation is one of the main features of the tumor microenvironment and the connection between inflammation and cancer is now accepted as enabling some of the most relevant characteristics of cancer, including chemoresistance.

### CONSTITUTIVE ACTIVATION OF NF-κB AS A TUMOR CELL-AUTONOMOUS MECHANISMS OF RESISTANCE

The ability of certain cancers to resist the cytotoxic effects of cancer chemotherapy appears to be closely connected to alterations in key pathways involved in cell-cycle checkpoint control and, most importantly, apoptosis.

Several autocrine and/or paracrine pro-inflammatory factors as well as pro-apoptotic stimuli – including chemotherapy and radiotherapy – lead to the activation of different transcription factors involved in apoptosis control. Nuclear Factor κB (NF-κB) is the most relevant of these transcription factors by representing a key mechanistic link between inflammation and cancer chemoresistance (Melisi and Chiao, 2007).

NF-κB aberrant activation plays a key role in the initiation and promotion of cancer by contributing to cell proliferation, angiogenesis and stimulation of invasion/metastasis. In particular, NF-κB activation can potently suppress cell death pathways, since it activates expression of several anti-apoptotic genes, including some *Bcl-2* family members, TNF receptor- associated factor 1 (TRAF1) and TRAF2, and c-IAP1 and c-IAP2 (Ben-Neriah and Karin, 2011). Because the cytotoxicity of chemotherapeutic agents is attributed largely to apoptosis, the activation of NF-κB can effectively suppress the apoptotic potential of chemotherapeutic agents, thus contributing a crucial obstacle to effective treatment of pancreatic cancer.

The initial evidences for the constitutive activation of NF-κB in pancreatic cancer were provided by seminal studies led by the research group of Paul Chiao at the MD Anderson Cancer Center. They firstly reported a constitutive activation of NF-κB signaling in 14 out of 20 pancreatic adenocarcinomas and in 9 out of 11 human pancreatic tumor cell lines (Wang et al., 1999). In a larger cohort of non-malignant and malignant pancreatic specimens, nuclear RelA staining was detected in 57% of pancreatic cancer samples. By contrast, RelA was detected in the cytoplasm of benign ducts from 96% patients. However, nuclear RelA staining was observed in a minority only (26%) of these benign ducts (Vimalachandran et al., 2005). In a different series of 82 pancreatic adenocarcinomas a strong cytoplasmic or nuclear expression of RelA/p65 was observed in 42 and 37 samples, respectively. High cytoplasmic and nuclear expression of RelA/p65 had negative prognostic impact with 2-year survival rates for patients without cytoplasmic or nuclear RelA/p65 positivity of 41 and 40% and rates for patients with strong cytoplasmic or nuclear RelA/p65 expression of 22 and 20%, respectively (Weichert et al., 2007).

Constitutive activation of NF-κB in pancreatic cancer seems to be not primarily determined by mutations of genes involved in its regulation, but rather by pro-inflammatory cytokines autocrine loops. interleukin-1α (IL-1α) and IL-1β are between the most potent cytokines that primarily affects inflammation, immunity and hematopoiesis (Dinarello, 1996; Apte et al., 2006). Niu et al. (2004) recently demonstrated that autocrine secretion of IL-1α, but not IL-1β, primarily induced by activator protein-1 (AP-1) activity, leads to the activation of NF-κB in metastatic pancreatic cancer cell lines but not in non-metastatic ones. In turn, NF-κB activation induces expression of IL-1α initiating the formation of a positive feedback loop and establishing a mechanism for the constitutive NF-κB activation in this disease. This autocrine secretion of IL-1α induced in turn a metastatic behavior *in vivo* as demonstrated by the higher incidence of liver metastases and ascites in an orthotopic mouse model (Melisi et al., 2009). More recently, Ling et al. (2012) generated a mutant mouse strain with pancreas-specific expression of KrasG12D and inactivation of IKK2/β demonstrating that NF-κB activity is required for oncogenic Kras-induced pancreatic cancer. Kras (G12D)-induced AP-1 transcription induced IL-1α, which, in turn, activates NF-κB and its target genes IL-1α and p62, to initiate an IL-1α/p62 feedforward loops for inducing and sustaining NF-κB activity. IL-1α overexpression correlated with Kras mutation, NF-κB activity, and poor survival in pancreatic cancer patients.

Several studies demonstrated that the resistance of pancreatic carcinoma cells to chemotherapy is indeed due to their constitutive NF-κB activity rather than a transient induction of NF-κB by different anti-cancer drugs (Arlt et al., 2001). In pancreatic cancer cell lines the uptake or intracellular targeting of gemcitabine was not altered, thus excluding a failure of drug uptake or metabolism as being responsible for the resistance of these cell lines against gemcitabine. Thus, the mechanism by which these cell lines are able to survive gemcitabine treatment only relies on an elevated basal NF-κB activity (Arlt et al., 2003). A complete inhibition of NF-κB activation by blocking degradation of both IκBα and IκBβ proteins is critical in order to suppress a potential NF-κB feedback reactivation induced by various anti-cancer agents, and in turn sensitize pancreatic cancer cell lines to apoptosis induced by these drugs (Dong et al., 2002).

Because of the experimental findings that inhibition of NF-κB alone or in combination with cancer therapeutic agents induces tumor cell death or inhibits tumor growth, NF-κB inhibition become an attractive strategy for novel chemopreventive and chemotherapeutic approaches. A large number of compounds with a putative NF-κB inhibiting activity have been identified, including a variety of natural and synthetic molecules, antioxidants, kinase inhibitors, peptides, and small molecules. However, direct targeting of NF-κB for cancer therapy still faces enormous challenges and most of these strategies remain thus far confined to the pre-clinical stage and very few of them have entered clinical trials for pancreatic cancer therapy (Carbone and Melisi, 2012).

In last years, we focused our efforts to identify cytosolic mediators of the activation of NF-κB that could represent relevant therapeutic targets for the inhibition of this pathway. Transforming growth factor-β (TGF-β)-activated kinase 1 (TAK1, also called MAP3K7) is a serine/threonine kinase in the mitogen-activated protein kinase kinase kinase (MAP3K) family. In the last decade, TAK1 has been clearly demonstrated to be a key player in the inflammatory responses and cell survival control, as it integrates signals from various cytokines – including IL-1, TGF-β, and toll-like receptors (TLRs) – controlling, in turn, the activation of different transcription factors, including AP-1, and NF-κB (Sakurai, 2012). In this regard, TAK1 represents a strong candidate to be the missing link between a proinflammatory tumor microenvironment and the resistance of cancer cells to the apoptotic potential of chemotherapeutic agents. We have recently demonstrated that silencing the expression or targeting the activity of TAK1 dramatically suppresses the DNA-binding activity of NF-κB leading to a proapoptotic phenotype in pancreatic cancer cells and in turn to their statistically significantly higher sensitivity to the anti-tumor activity of chemotherapeutic drugs. More importantly, we demonstrated that targeting the kinase activity of TAK1 with the selective inhibitor LYTAK1 enhanced the anti-tumor activity of chemotherapeutic drugs *in vivo* in an orthotopic model of pancreatic cancer, indicating that the inhibition of the kinase activity of TAK1 could be a valid approach to reverting the intrinsic chemoresistance of pancreatic cancer (Melisi et al., 2011). Targeting TAK1 as a non-redundant cytosolic mediator of the activation of NF-κB could represent a novel approach to modulate the intrinsic and acquired chemoresistance and make a difference in pancreatic cancer addicted to the activity of this transcription factor.

### EXTRINSIC MECHANISMS OF RESISTANCE: THE ROLE OF TUMOR STROMA

Beside tumor-cell autonomous mechanisms of resistance, recent data suggest an important role for tumor microenvironment in the development and maintenance of resistance to classic chemotherapeutic and targeted therapies. Tumor stroma, in particular, seems to significantly limit the ability of drugs to penetrate and reach tumor cells at therapeutically relevant concentrations.

To reach all viable cells in the tumor, anti-cancer drugs must be delivered efficiently through the tumor vasculature, cross the vessel wall, and traverse the tumor tissue (Tredan et al., 2007). Compared with normal tissues, the tumor stroma is associated with an altered extracellular matrix and an increased number of fibroblasts that synthesize growth factors, chemokines, and adhesion molecules. Moreover, tumor stroma is characterized by an increased interstitial fluid pressure, which leads to a decreased uptake of drugs into the tumor, lowering thus their therapeutic efficiency (Heldin et al., 2004).

Pancreatic cancer is one of the human tumors most rich in stroma with a dynamic assortment of extracellular matrix components and non-neoplastic cells including fibroblastic, vascular, and immune cells (Feig et al., 2012). One of the most relevant signaling pathways involved in microenvironment-related chemotherapy resistance in pancreatic cancer is the hedgehog (HH) pathway. HH signaling between tumor and stromal cells brings about a desmoplastic reaction where the stromal fibroblasts secrete collagen in higher amounts and result in fibrosis of the surrounding stromal tissue. Studies using genetically engineered mouse models of pancreatic cancer highlighted the role of HH pathway in drug resistance by inducing a desmoplastic reaction, decreasing the mean vascular density around the tumor tissue and thus the delivery of gemcitabine to the tumor (Olive et al., 2009). Use of HH signaling targeting agents like cyclopamine derivatives inhibited transcriptional activation of target genes, preventing in turn development of desmoplastic matrix and increasing intratumoral perfusion and drug delivery.

Despite encouraging results from phase I studies, HH pathway inhibitor IPI-926 (saridegib) was not beneficial when added to gemcitabine in a randomized phase II study in patients with metastatic pancreatic cancer, showing a disappointing outcome with difference in patient survival favoring the placebo plus gemcitabine arm. A recent phase Ib study presented at the 2012 ASCO annual meeting proposed the combination of IPI-926 with a more intensive chemotherapy platform (mFOLFIRINOX) in patients with advanced pancreatic cancer, showing good safety and efficacy data (Ko et al., 2012).

Different agents with anti-fibrotic effects have shown promising results in pre-clinical models of pancreatic cancer (Erkan et al., 2012). Hyaluronic acid (HA) demonstrated to be one of the most relevant matrix determinant of vascular collapse in pancreatic cancer models. The systemic administration of an enzymatic agent PEGPH20 that ablates stromal HA from murine pancreatic cancer models normalized interstitial fluid pressures, and re-expanded the microvasculature. Removing these barriers permitted higher concentrations of chemotherapy to reach the tumor, resulting in improved survival and revealing an unappreciated sensitivity of the disease to conventional cytotoxic agents like gemcitabine (Provenzano et al., 2012).

Furthermore, the pre-clinical utility of PEGPH20 in combination with gemcitabine was also assessed by short-term and survival studies in a genetically engineered mouse model. In these models PEGPH20 not only rapidly induced the re-expansion of blood vessels, but it also triggered fenestrations and interendothelial junctional gaps in tumor endothelia and promoted a tumor-specific increase in macromolecular permeability, which results in intratumoral accumulation of two chemotherapeutic agents, doxorubicin, and gemcitabine (Jacobetz et al., 2013).

The tumor microenvironment in pancreatic cancer is largely immunosuppressive, restraining anti-tumor immunity. A strategy proposed to overcome stromal desmoplasia is depletion of tumor-associated fibroblasts by using CD40 agonists. Because CD40 activation can reverse immune suppression and drive anti-tumor T cell responses, the combination of an agonist CD40 antibody with gemcitabine chemotherapy was tested in a small cohort of patients with advanded pancreatic cancer. CD40-activated macrophages infiltrated the tumor and became not only tumoricidal but also facilitated the depletion of tumor stroma, inducing tumor regressions in some patients as well as in a genetically engineered mouse model of pancreatic cancer (Beatty et al., 2012).

Beside the impairment in drug delivery, several studies highlighted an important active role for stromal cellular components. Among them, pancreatic cancer cells promote the activation, proliferation and motility of a particular subpopulation of stromal cells, pancreatic stellate cells (PSCs). Activated PSCs can transdifferentiate into a myofibroblast-like phenotype and secrete extra cellular matrix proteins and factors which mediate tumor growth, invasion, metastasis, and resistance to chemotherapy (Vonlaufen et al., 2008; Erkan, 2012). Similarly, recent studies observed reduced response to gemcitabine of pancreatic cancer cells in co-culture systems with stromal cells (Straussman et al., 2012), as well as in pancreatic cancer cells treated with stromal cell conditioned medium (Hwang et al., 2008).

Overall, these studies suggest that stromal cells within tumor microenvironment promote chemoresistance and represent a critical constituent of pancreatic cancer that should be critically evaluated for an optimal therapeutic development.

## PANCREATIC CANCER RESISTANCE TO ANTIANGIOGENIC AGENTS

Tumor angiogenesis represents a hallmark of cancer (Hanahan and Weinberg, 2011), and thus multiple approaches have been pursued to block or at least reduce the extent of aberrant vessel formation. One prominent player in this process is the vascular endothelial growth factor (VEGF) signaling pathway (Tortora et al., 2004). VEGF targeting agents are particularly attractive because of the multiple roles of VEGF in tumor biology not only on tumor vasculature but also on tumor cell proliferation (Ciardiello et al., 2004; Ellis and Hicklin, 2008). In pancreatic carcinoma, overexpression of VEGF and its receptors has been associated with poor prognosis and increased metastatic potential (Seo et al., 2000). The expression of VEGF correlates with advanced stage and predicts early recurrence after resection (Niedergethmann et al., 2002).

The first anti-angiogenic drug to be approved for the treatment of several metastatic cancers was bevacizumab, a humanized monoclonal VEGF-neutralizing antibody. In a randomized phase II clinical trial, bevacizumab showed promising activity in combination with gemcitabine in patients with advanced pancreatic cancer (Kindler et al., 2005). However, this activity was not confirmed in the CALGB study 80303 phase III clinical trial comparing gemcitabine alone to gemcitabine with bevacizumab (Kindler et al., 2010). In the phase III AviTA trial, evaluating the addition of bevacizumab to gemcitabine plus erlotinib, bevacizumab prolonged patients’ progression free survival, but not overall survival (Van Cutsem et al., 2009). Similarly, other anti-angiogenic agents – Aflibercept, a VEGF-trap antibody, or Axitinib, a VEGFR small molecule inhibitor, did not prolong patients’ survival over gemcitabine as single agent treatment in clinical trials for the treatment of metastatic pancreatic cancer (Ebos and Kerbel, 2011).

The current view explaining resistance to anti-angiogenic therapies is based on two modalities: an intrinsic resistance to the drug when tumors do not respond *ab initio*, and an evasive resistance for those tumors that recur after a short period of response (Bergers and Hanahan, 2008).

Some of the molecular bases of these two resistance modalities are shared, being them a peculiar characteristic of the tumor cells or caused by anti-angiogenic drug-derived hypoxia in the stroma. Common explanations for the refractoriness to anti-angiogenic therapies can be: (a) redundancy of proangiogenic stimuli; (b) recruitment in the tumor environment of myeloid cells producing inflammatory and proangiogenic cytokines (Shojaei et al., 2007); and (c) absence of neo-angiogenesis when tumors take advantage of pre-existing normal vessels. These mechanisms of resistance could co-exist in a certain tumor type.

It has been proposed that failure of anti-angiogenic therapy in the case of pancreatic cancer is more likely due to an intrinsic independency from the vascular related effects of VEGF. Indeed, pancreatic cancers are hypovascularized and feature a desmoplastic stroma. Besides its histological setting, pancreatic cancer frequently features p53 mutations which confer the ability to survive even in highly hypoxic condition. The reason for the lack of a sustained angiogenesis program in pancreatic cancer, a striking difference compared to other solid tumors, is unknown. Despite the lack of vascularization though, pancreatic cancer are still good candidates to the treatment with anti-angiogenic drugs. Indeed, late stage pancreatic cancer patients often present with high volume of ascites, so normalization of the balance between vascular permeability and lymphatic drainage through anti-angiogenic drugs could reduce the effect of excessive tumor burden which also prevents the activity of chemotherapy.

Data from our lab (Carbone et al., 2011) are in support of the hypothesis that actually the resistance to anti-VEGF therapy is mediated by tumor cells autonomous secretion of chemokines that have both paracrine and autocrine effects. Gene expression profiles of our bevacizumab resistant model highlighted an increased production of chemokines that are important for the attraction of myeloid cells and mobilization of their precursors from bone marrow. These cells have a role both in promotion of tumor angiogenesis (Murdoch et al., 2008) as well as in maintenance of an inflammatory environment that sustains tumor progression (Coussens et al., 2013). These observations can explain the lack of efficacy of anti-VEGF agents since the positive effect of blood supply reduction is counteracted by indirect pro-angiogenic and pro-inflammatory effect, thus suggesting the possibility to add anti-inflammatory agents in the therapy of pancreatic cancer. In this scenario the evaluation of targeted therapies that block inflammatory pathways activated in the context of anti-angiogenic treatment would be important to select candidate drugs to be tested in the clinic.

Another caveat against the use of anti-angiogenic therapy is the emerging possibility of a more aggressive phenotype in anti-VEGF treated cells and increased frequencies of metastatic lesions (Loges et al., 2009). In our model we observed epithelial mesenchymal transition (EMT) characteristic changes in anti-VEGF resistant cells which could account for an increased metastasis rate. Bevacizumab resistant cells featured increased motility and invasion compared to sensitive cells, up-regulated expression of Zeb1, Zeb2, and SMAD3 genes, that are components of an EMT transcriptional signature. Additionally E-cadherin epithelial marker was strongly down-regulated as opposed to up-regulation of Vimentin mesenchymal marker. Differently, a recent study performed in genetically engineered mouse models of solid tumors, among them KRAS driven pancreatic cancer, did not confirm an increased metastases frequency in anti-VEGF antibody treated mice, neither in monotherapy nor in combined therapy with gemcitabine (Singh et al., 2012). This topic is controversial since different groups report distinct results, also depending on the anti-VEGF approach. Clarifying this subject is particularly important in light of antiangiogenic approaches in pancreatic cancer and it should be addressed whether anti-VEGF antiangiogenic therapy can determine a more invasive phenotype only when targeting VEGFR by receptor tyrosine kinase inhibitors or it could also be a drawback of the use monoclonal antibodies directed against VEGF.

## CONCLUSION

Pancreatic cancer resistance to chemotherapeutic and anti-angiogenic agents is attributable to intrinsic tumor cell characteristics as well as to extrinsic factors, such as properties of tumor microenvironment.

Constitutive activation of NF-κB in pancreatic cancer represents the main intrinsic mechanisms of resistance due to suppression of apoptosis. Experimental pre-clinical evidences support the potential efficacy of anti-NF-κB strategies, but to date there are no direct NF-κB inhibitors available for patients treatment. However, the possibility to inhibit NF-κB seems to be pursued indirectly by using inhibitors of mediators for NF-κB activation. In this regard the use of TAK1 inhibitor has proven successful to overcome chemoresistance in pancreatic cancer models.

Extrinsic regulators of chemoresistance are represented by cellular components of the stroma, and the extracellular matrix that they produce. Abundant desmoplastic stroma may represent a barrier for chemotherapetic drugs delivering to the tumor. However, approaches to inhibit the signaling pathways that regulate collagen secretion by fibroblast, such as HH signaling inhibitors did not reach advanced stage of clinical trials so far.

Resistance to anti-angiogenic therapy in pancreatic cancer occurs through secretion of proinflammatory cytokines that recruit myeloid cells, which in turn concur to sustain inflammation. The outcome of persistent inflammation may be not only tumor resistance but rather recurrence characterized by a more aggressive phenotype.

In conclusion, we have summarized the main aspects in the current knowledge about drug resistance in pancreatic cancer. Further researches to clarify the main regulators of these mechanisms will be important to provide the rationale to develop effective combination treatment strategies for pancreatic cancer patients.

## Conflict of Interest Statement

The authors declare that the research was conducted in the absence of any commercial or financial relationships that could be construed as a potential conflict of interest.
